# Assessment of Executive and Cognitive Functions in Children with Restless Sleep Disorder: A Pilot Study

**DOI:** 10.3390/brainsci12101289

**Published:** 2022-09-24

**Authors:** Lourdes M. DelRosso, German Vega-Flores, Raffaele Ferri, Maria P. Mogavero, Adele Diamond

**Affiliations:** 1 Center for Clinical and Translational Research, University of Washington, Seattle Children’s Hospital, Seattle, WA 98105, USA; 2 Ciencias de la Salud, Universidad Internacional de Valencia, 46002 Valencia, Spain; 3 Educación, Universidad Internacional de La Rioja, 26006 Logroño, Spain; 4 Sleep Research Centre, Oasi Research Institute—IRCCS, 94018 Troina, Italy; 5 Institute of Molecular Bioimaging and Physiology, National Research Council, Segrate, 20054 Milan, Italy; 6 Sleep Disorders Center, Division of Neuroscience, San Raffaele Scientific Institute, 20127 Milan, Italy; 7 Developmental Cognitive Neuroscience Program, Department of Psychiatry, The University of British Columbia, Vancouver, BC V6T 1Z4, Canada

**Keywords:** restless sleep disorder, selective attention, pediatrics, executive functions

## Abstract

Restless sleep disorder affects children and is characterized by frequent nocturnal movements, iron deficiency, and daytime symptoms such as poor school performance or behavioral problems. Although sleep parameters have been thoroughly studied and daytime sleepiness has been previously assessed, neurocognitive and executive functions have not. In this study, we evaluated neurocognitive functions in a group of 13 children diagnosed with restless sleep disorder using the National Institute of Health Toolbox (NIH toolbox). The mean age was 10.62 (S.D. 2.785). Among them, seven were male and six were female. The fully corrected T-scores (adjusted for demographic variables: age, ethnicity, and education level) showed the lowest values for the Flanker test (selective attention) and dimensional change card sorting test (cognitive flexibility and inhibitory control), with a very large effect size vs. the corresponding expected frequencies. For all the other tests, the average scores were 50; however, individual children scored low on pattern recognition and two composite scores (fluid and total). In conclusion, these data support the fact that cognitive functions are affected in children with restless sleep disorder, especially selective attention. Clinicians must recognize sleep disorders and daytime impairment in order to promptly intervene and prevent cognitive impairments.

## 1. Introduction

Good sleep quality is important for healthy growth, development, and cognition [1]. Decades of research have demonstrated the importance adequate sleep time. In fact, the American Academy of Sleep Medicine (AASM) has published an expert consensus guideline recommending the hours that children should sleep on the basis of age (children 3–5 years: 10–13 h; 6–12 years: 9–12 h; teenagers 13–18 years: 8–10 h) [2]. Sleeping the right amount of time helps children and adolescents avoid the consequences of sleep deprivation, which include daytime sleepiness, hyperactivity, and attention problems, among others [3]. 

Sleep disorders can affect both quantity and quality of sleep; however, when compared with sleep quantity, sleep quality has not been thoroughly studied. Depending on the symptoms and presentation, sleep disorders are divided in six main categories: insomnia, parasomnia, hypersomnia, circadian rhythm disorders, sleep disordered breathing, and movement disorders [4]. In the last decade, sleep medicine has advanced in knowledge of the consequences associated with sleep disorders in relationship with health, quality of life, behavior, cognition, and executive function, particularly in adults [5,6]. Unfortunately, studies demonstrating the daytime consequences of sleep disorders, particularly in cognition and executive function, in children are sparse [7,8]. Sleep-related movement disorders include restless legs syndrome, periodic limb movement disorder, bruxism, rhythmic movement disorder, and restless sleep disorder (RSD) [9]. In some of these disorders, such as in RSD, the amount of sleep is not affected but the quality of sleep is compromised, contributing to daytime symptoms such as sleepiness and fatigue [10]. RSD has been identified in children aged 6–18 years [11] and is manifested mainly by frequent movements during sleep. The diagnostic criteria of RSD include parental complaints of restless sleep manifested by frequent large muscle movements during sleep; repositioning or movements occurring throughout the night; and restless sleep associated with daytime symptoms of sleepiness, hyperactivity, or behavioral or cognitive problems [11,12]. Polysomnography is required for the diagnosis since objective findings of frequent body movements must be demonstrated; in fact, the diagnosis of RSD requires a sleep study to rule out other sleep disorders and to demonstrate a large body movement index of at least five movements per hour [11,13,14]. RSD is found in 7.7% of children referred to sleep centers, a prevalence around that of insomnia (7.3%), and below the prevalence of restless legs syndrome (10.3%) [14]. The pathophysiology of RSD has not been completely elucidated, but some postulated theories include sleep instability, sympathetic activation, and iron deficiency [12,15,16]. Treatment with oral or intravenous iron has been shown to improve the symptoms of RSD [17].

Most neuropsychological evaluations of executive and cognitive functions have been carried out in adults with sleep disorders [18]. It is clear that adults with obstructive sleep apnea have impaired non-verbal reasoning [19], attention, visual and verbal memory [20], and visuospatial constructional abilities [5,6]. The same results have not been as robust in children with obstructive sleep apnea. Children’s scores decrease but remain within the expected range for their ages [21]. This is particularly important because it is currently not known how long obstructive sleep apnea has to be present to affect neurocognitive pathways in developing children.

When evaluating daytime impairment, it is important to differentiate cognitive functions from executive functions. Cognitive functions are those abilities that allow us to carry out tasks and include memory, language, and attention, while executive functions are necessary for the cognitive control of behavior, such as selecting adequate behaviors for the appropriate time, as well as switching behaviors, if needed. There are three main core executive functions: inhibitory control, working memory, and cognitive flexibility; from these, higher-order executive functions are built: reasoning, planning, and problem solving [22]. Inhibitory control is the ability to focus attention, actions, thoughts, and emotions, resisting internal or external distractions and providing a considered response rather than an impulsive one [23]. Working memory is the ability to hold information in the mind and work with it, such as using previously learned information to solve novel problems. Deficiencies in working memory can manifest as difficulty following instructions or a constant need for repetition [22]. Cognitive flexibility is the ability to adapt our responses to the demands of new requirements, which allows us to change strategies or see a situation from a different point of view [22]. 

Although cognitive and executive functions have been studied in children with other sleep disorders such as obstructive sleep apnea [24,25,26], they have not been studied in children with sleep-related movement disorders, such as children with RSD. In this pilot study, we aimed to study the presence and relationship between deficits in cognitive and executive functions in children with a diagnosis of RSD. The study had two specific aims: (a) to evaluate cognitive and executive functions in children with RSD, and (b) to determine if any cognitive or executive function is more affected in children with RSD than other cognitive or executive functions.

Our main hypothesis is that children with RSD will present with deficits in both cognitive and executive functions.

## 2. Materials and Methods

### 2.1. Subjects

Thirteen subjects were consecutively recruited from the sleep center at Seattle Children’s Hospital. Inclusion criteria were as follows: diagnosis of RSD with clinical and polysomnographic evaluation, and no history of recent infection or inflammation (past 2 months) with C-reactive protein <1. All children were attending an age-appropriate grade in school. Exclusion criteria were as follows: children younger than 6 or older than 18 years; children with syndromes, neurodevelopmental disorders, or inability to speak or express themselves; comorbidity with other medical, sleep, or psychiatric disorders; children who did not complete polysomnography; children who did not speak English; children taking medication that affects sleep or alertness. A board-certified pediatric sleep physician (LDR) evaluated all children with a complete intake history and physical exam, including complete chart reviews for comorbidities and medications. Diagnosis of RSD was based on published criteria [27].

### 2.2. Instruments

All parents filled out sleep diaries to ensure proper sleep time (no sleep deprivation) prior to neurocognitive testing. All children underwent polysomnography to rule out other sleep disorders and to diagnose RSD. Polysomnography was performed according to the AASM criteria [28], and data were recorded using the Sandman Elite Natus system, Middleton, WI 53562 USA. Parameters recorded included electroencephalogram (EEG: two frontal, two central, and two occipital channels, referred to the contralateral mastoid), electro-oculogram, electromyogram (EMG) of the submentalis muscle, EMG of the right and left tibialis anterior muscles, respiratory signals, effort signals for the thorax and abdomen, oximetry, capnography, a single-lead electrocardiogram, and video and audio recording. Calibrations were performed per routine standard by technicians. Epochs were scored by a certified sleep technologist and board-certified sleep physician according to the AASM criteria. RSD movements during sleep were scored according to the published criteria [29]. 

The NIH toolbox cognitive battery was used for this study [30]. This battery is designed to be administered to patients aged from 3 to 85 years. The cognitive battery evaluates executive functions, attention, memory, processing speed, and language with age-appropriate measures in 5 age groups: 3–4, 5–7, 8–12, 13–17, and 18 and older. All testing was conducted 2 h after awakening, on a Saturday. 

The NIH toolbox cognitive battery includes the following tests. The Dimensional Change card sorting test evaluates cognitive flexibility and inhibitory control in children [31]; the Flanker test assesses selective attention [30]; the Picture Sequence memory test evaluates episodic memory [32]; List Sorting assesses working memory [32]; Picture Vocabulary measures the language skill of receptive vocabulary [32]; Pattern Recognition measures processing speed [32]; and the Oral Reading test measures reading abilities.

Each test provides individual scores, performance scores, and composite scores. Normative data are provided in the NIH toolbox for performance and composite scores. 

The fully corrected T-score (with a mean of 50 and standard deviation of 10) compares the score of the test to the NIH toolbox normative sample, while adjusting for demographic variables (age, ethnicity, and education level). The cognition battery produces 3 composite scores, which are also given as T-scores: the fluid cognition composite score represents the ability to solve problems, act quickly, and encode new memories; the crystallized cognition composite score represents the accumulated experiences of verbal knowledge and skills; finally, the cognitive function composite score is considered a reliable overall measure of cognitive function. 

### 2.3. Statistics

Descriptive statistics were performed using SPSS Statistics 28 (IBM). Mean and standard deviation were calculated for age. Frequencies were calculated for sex and ethnicity. Means, range, and standard deviation were calculated for the performance T-scores and composite score. The comparison of the frequency of the observed normalized T-scores ≤50 and >50 vs. their expected frequencies was carried out by means of the chi-squared test, and the corresponding effect size φ was computed (φ = √χ^2^/n); a value of 0.1 is considered a small effect, 0.3 a medium effect, and 0.5 a large effect. Finally, the nonparametric Mann–Whitney test was used for independent data comparisons.

## 3. Results

**Figure 1:**
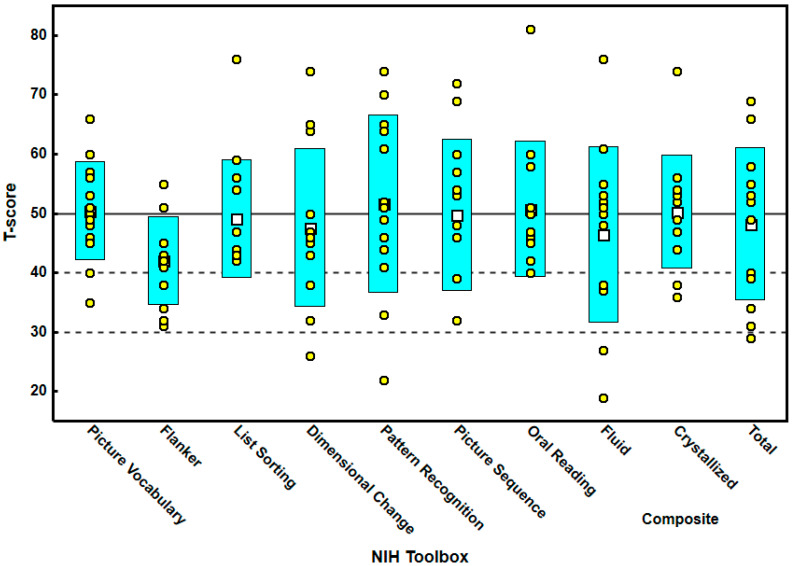
Box plot demonstrating the T-scores for all the tests in the cognitive battery of the NIH toolbox. Note that the Flanker test had the lowest scores. The data display shows the mean (white-filled squares), standard deviation (cyan-filled boxes), and individual data (yellow-filled circles).

Thirteen consecutive children were included in this case series. Their mean age was 10.62 (S.D. 2.785); seven were male and six were female. Nine were Caucasian, two were Latino, and two were Asian. The T-scores for all the tests are found in Table 1. It should be noted that the lowest score was the Flanker score with a mean score of 42, a minimum score of 31, and a maximum score of 55. For all the other tests, the average scores were close to 50. For T-scores, any score below 30 is less than 2 standard deviations from the mean. Figure 1 shows that some children scored below 30 on dimensional change, pattern recognition, and two composite scores (fluid and total). Figure 1 also demonstrates visually that the majority of children with RSD scored below the mean T-score of 50 for the Flanker test. 

Table 2 shows the comparison of the frequency of the observed normalized T-scores ≤50 and >50 vs. their expected frequencies. The chi-squared test did not disclose any significant difference (due to the small sample size) but only a tendency towards statistical significance for Flanker and dimensional change (*p* = 0.067 for both). However, the corresponding effect size φ was found to be very large for these two scores and, in addition, an effect size of 0.474 was found for list sorting, indicating an almost large effect size (a value of 0.5 is considered a large effect).

Finally, no statistically significant difference between T-scores obtained in males and females was found, and all comparisons showed a small effect size.

## 4. Discussion

This study shows that children with RSD tend to score below the average in selective attention, which is assessed by the Flanker test (Figure 1). These findings bring insight into the daytime function and symptoms of children with RSD. Just like previous studies on executive and cognitive function in children with sleep disorders, most of the findings in the other tests were within normal levels, although in some cases below 1 or 1.5 standard deviations [33]. Studies that have used uncorrected scores have found that, when the scores were adjusted for age or socioeconomic status, differences were even smaller [33]; therefore, we decided to use adjusted T-scores for our current study. 

This is the first study that has assessed neurocognitive function in children with RSD, highlighting the importance of the contribution of poor sleep quality to daytime symptoms beyond daytime sleepiness, in this case, selective attention. 

Our results show that there may be patterns of neurocognitive weakness that may be specific to RSD, particularly in children with lower scores in attention. The NIH toolbox has been used to assess neurocognitive function in children and adolescents with other conditions and has also found pattern deficiencies that seem to be condition specific. For instance, adolescents and young adults with autism spectrum have demonstrated that, among the other tests, the lowest scores have been found in pattern comparison processing speed [34], findings that have been corroborated in adults [35]. Slower processing speed can contribute to some aspects of psychosocial functioning found in patients with autism. It is worth mentioning that the lowest scores in these studies were between the mean and 1 standard deviation below [35]. In contrast to these findings, adolescents with Tourette’s syndrome did not show average results below the 50th percentile, with the lowest scores in list sorting working memory [34], which have also been corroborated in other studies [36]. These studies exemplify that the neurocognitive batteries can show discrete patterns of subtle deficits in areas that can be disorder specific. They can also help differentiate behaviors in children. For instance, with the suggestion that attention is lower in children with RSD, we can understand that school performance and behavior may not be secondary to impulsivity, distraction, memory, or maybe sleepiness. This unique profile, if confirmed by larger, controlled studies, can aid in the identification of daytime symptoms and diagnosis of RSD. Identifying these subtle weaknesses can also provide therapeutic guidance in the future. In a study by Chervin et al. [37], children with mild-to-moderate sleep disordered breathing had deficits in attention that improved a year after adenotonsillectomy. Studies have shown that children with RSD subjectively improve in sleep and daytime symptoms after treatment with iron supplementation [17]. In this sense, symptoms suspicious of attention deficit in children with RSD should be explored with polysomnography to assess if RSD criteria are fulfilled, and prompt treatment with iron supplementation should be instituted. If symptoms persist, assessment for comorbid conditions that affect attention (ADHD) should be pursued.

The neurobiology of selective attention has been a focus of research in particular for children with attention deficit/hyperactivity disorder (ADHD) [38]. Neuroimaging studies have pointed to neural networks associated with selective attention found in the pre-striate areas, concerned with basic visual processing [39]. Positron emission tomography studies (PET) have also shown enhancement within different visual associative regions of the brain, with attention to different attributes, demonstrating the interaction of other brain areas for different stimuli, such as intraparietal sulcus for speed; collateral sulcus and dorsolateral occipital cortex for color; collateral sulcus, fusiform and parahippocampal gyri, and superior temporal sulcus for shape [40]. During childhood, these neuronal networks are active and developing. It is of utmost importance to identify contributors to disruption in these networks during the early years of childhood to provide prompt intervention. In fact, studies on sleep deprivation have corroborated the impact on neuronal networks associated with attention and alertness [41].

Vigilance and wakefulness are two basic processes over which executive and cognitive functions develop. The degree of alertness varies during the day, affected mainly by circadian influences [42]. However, alertness can also be influenced by the amount and quality of sleep [43]. Studies have evaluated the impact of sleep deprivation on sustained attention and executive functions [44]. Sleep deprivation impairs vigilant attention and processing speed [45] and has also been shown to impair sustained attention, both in children with ADHD and with normal controls [46]. Previous studies have shown that children with RSD do not present with symptoms of insomnia or significant sleep deprivation. In fact they do not have difficulty falling asleep or extended nocturnal awakenings [12]. Sleep disruption in RSD, however, is likely to be secondary to microstructural interruptions during sleep with higher sleep instability [15], possibly associated with the frequent movements and repositioning through the night. In this respect, it is important to emphasize that sleep microstructure has been reported to be correlated with cognitive processing and next-day cognitive performance [47,48,49]; thus, it might also have a role in cognitive changes in RSD. The microstructural sleep impairment in children with RSD is further supported by an increased sympathetic activation during sleep, which was found during N3 and REM sleep in particular through analyzing heart rate variability [16]. 

Regarding other sleep-related movement disorders, a study in patients with restless leg syndrome has shown deficiencies in verbal fluency, short attention span, and inhibitory control [50]. These findings were further confirmed in patients with restless leg syndrome and poor sleep quality [51]. Other studies using the Stroop and trail-making tests did not identify differences between patients with restless leg syndrome and controls [52], pointing out the possibility that other influencing factors, such as disease duration, severity of symptoms, or degree of sleep disruption, might contribute to the daytime impairment. 

Although there is a scarcity of publications evaluating cognitive functions and executive functions in children with sleep-related movement disorders, we hypothesize that sleep disruption contributes to daytime cognitive effects. Another important point in our study is the fact that some children with RSD presented with low scores in tests that assess processing speed, inhibitory control, and cognitive flexibility. These executive functions are crucial for early success in school, helping with organizational skills, reasoning, and cognitive development [53]. 

Limitations of this study include the small number of patients and the absence of a normal control group due to COVID-19 restrictions in our clinic; moreover, patients were recruited by a single center and only one, although complete, strongly validated and reliable neuropsychological assessment tool was used. These limitations preclude the generalization of the results reported above; however, the exploratory and observational nature of this study establishes a starting dataset for more detailed and conclusive future research. Indeed, larger, more diverse populations need to be studied to corroborate the findings presented here.

## 5. Conclusions

This exploratory study suggests that more detailed studies are needed to assess sleep disorders, including RSD, in children with restless sleep and daytime symptoms. It is the authors’ opinion that children should be periodically assessed for executive and cognitive function to optimize areas that can contribute to overall health and normal development. Furthermore, children suspected of having disturbances in attention should be evaluated for sleep disorders.

A future direction for research includes assessment of neurocognitive function before and after iron treatment, larger samples of children with RSD, and longitudinal research on the developmental trajectory of neuropsychological deficits among children and adolescents with RSD as they grow into adulthood.

## Figures and Tables

**Table 1:** T-scores for all the tests in the NIH toolbox including composite scores.

	Min	Max	Mean	S.D.
Picture vocabulary	32	66	50.23	8.757
Flanker	31	55	42.08	7.331
List sorting	42	76	48.77	9.808
Dimensional change	26	74	47.92	13.131
Pattern recognition	22	74	51.85	14.781
Picture sequence	31	72	49.92	12.939
Oral reading	40	81	50.85	11.371
Composite fluid	19	76	46.46	14.858
Composite crystallized	36	74	50.31	9.517
Composite cognitive	29	69	48.23	12.788

Min = minimum; Max = maximum; S.D. = standard deviation.

**Table 2:** Frequency of T-scores for all the tests in the NIH toolbox equal to or smaller than 50.

	T-Score ≤ 50	T-Score > 50	Chi-Squared	*p* =	Effect Size φ
Picture vocabulary	7	6	0.07	0.791	0.019
Flanker	10	3	3.35	0.067	0.929
List sorting	9	4	1.71	0.191	0.474
Dimensional change	10	3	3.35	0.067	0.929
Pattern recognition	6	7	0.07	0.791	0.019
Picture sequence	7	6	0.07	0.791	0.019
Oral reading	8	5	0.61	0.435	0.169
Composite fluid	7	6	0.07	0.791	0.019
Composite crystallized	6	7	0.07	0.791	0.019
Composite cognitive	6	7	0.07	0.791	0.019

## Data Availability

The data presented in this study are available upon reasonable request to L.M.D.
